# Barriers to uptake of harm reduction techniques for GBMSM who use drugs in night-clubs and sex-on-premises venues in London and the Southeast: a mixed-methods, qualitative study

**DOI:** 10.1186/s12954-025-01159-2

**Published:** 2025-02-01

**Authors:** Stephen Naulls, K. Oniti, J. Eccles, J. M. Stone

**Affiliations:** 1 Department of Clinical Neuroscience, Brighton and Sussex Medical School, Brighton, UK; 2 Department of Psychiatry, Sussex Partnership NHS Trust, Worthing, UK; 3 School of Primary Care and Public Health, Faculty of Medicine, Imperial College School of Medicine, London, UK; 4 Institute of Global Health Innovation, Department of Surgery & Cancer, Imperial College School of Medicine, London, UK; 5 Sussex Neurodevelopmental Service, Sussex Partnership NHS Trust, Brighton, UK

**Keywords:** LGBTQIA+, GBMSM, CHEMSEX, Sexualised drug use, People who use drugs, Harm reduction, Addiction

## Abstract

**Background:**

Drug-related harm is a significant public health concern in the UK, particularly among underserved groups such as gay, bisexual, and other men who have sex with men (GBMSM). This study explores the role of night-time venues (for example night clubs or sex-on-premises venues) in promoting harm reduction strategies for GBMSM who use drugs, highlighting unique challenges within these spaces.

**Methods:**

The study used a mixed-methods approach, including an online survey (*n* = 53) and semi-structured interviews (*n* = 8). Participants included GBMSM with lived experience of substance use in night-time venues, as well as those providing support to this population. Data was collected through a Likert-scale survey and thematic analysis of qualitative responses.

**Results:**

Findings reveal dissatisfaction among survey respondents about the level of support for harm reduction provided by night-time venues, which are perceived as inconsistent in their approach towards substance use. The study also identifies economic and legal barriers faced by venues that prevent the endorsement of harm reduction techniques.

**Conclusions:**

Addressing these barriers could transform night-time venues into effective sites for harm reduction, particularly by targeting “afters” culture (the phenomenon where club-goers will return to a residential setting and continue substance use for prolonged periods ‘after’ the night-time venue closes or the event ends) and promoting safer practices. This research suggests that coordinated efforts with local government and policy reform are crucial to fostering safer environments for GBMSM.

**Supplementary Information:**

The online version contains supplementary material available at 10.1186/s12954-025-01159-2.

## Introduction and background

Drug-related harm is an escalating public health concern in the UK, particularly among underserved groups such as the LGBTQIA + community [1]. LGBTQIA + people are disproportionately affected by substance use and its associated harms, including higher rates of dependency, mental health issues, and drug-related mortality, compared to their heterosexual and cisgender counterparts [2–6]. Within this, gay, bisexual, and other men who have sex with men (GBMSM) face distinct challenges in relation to substance use and associated harms [7, 8]. This disparity is partly driven by a range of factors including minority stress, experiences of discrimination, and limited access to culturally competent or identity-affirming healthcare services [8, 9].

The substance use landscape within the LGBTQIA + community has specific unique characteristics, with distinct patterns of drug use that differ from the general population [6, 10]. Many LGBTQIA + individuals choose to attend specific LGBTQIA + venues, often in response to historic discrimination in conventional venues and a desire to find ‘safe spaces’ [11]. Among GBMSM, one notable trend is the increasing prevalence of a specific type of ‘sexualised drug use’ named ‘chemsex’, which involves using substances including GHB/GBL, crystal methamphetamine, and mephedrone to enhance sexual experiences [12–15]. Chemsex carries specific health risks, including a high likelihood of overdose, sexually transmitted infections, and severe mental health impacts [16, 17]. Consequently, much of the recent research and intervention efforts have focused on understanding and mitigating the harms associated with chemsex, particularly within the GBMSM community in urban centres such as London [18–20].

However, this focus on chemsex, while important, has overshadowed broader issues related to substance use in LGBTQIA + spaces. Many GBMSM also engage in non-sexual substance use, particularly with ‘party drugs’. A party drug (or club drug) refers to a substance used recreationally in social or party settings, such as nightclubs, music festivals, or other gatherings. These drugs are often used for their stimulant, euphoric, or hallucinogenic effects, which may enhance sociability, energy, or sensory experiences [21]. Examples of such ‘party drugs’ include MDMA, cocaine, and ketamine, which are prevalent in LGBTQIA + nightclubs and other nightlife venues [22–25]. The culture of substance use in these settings is typically normalised, possibly contributing to a range of harms, including dependency, mental health challenges, and risky behaviours. Despite these issues, there has been relatively little research on the broader phenomenon of party drug use within LGBTQIA + venues, where much of this activity takes place [22, 25].

Illicit drug use in night-clubs can also be linked directly to chemsex. Many LGBTQIA + nightclub events or raves have ‘darkrooms’ which are areas of the night-club where sexual activities are permitted; often whilst individuals are intoxicated [26, 27]. Moreover, ‘afters’ culture is prominent among LGBTQIA + users of party-drugs, including GBMSM, where people who use drugs (PWUD) in night-clubs will leave the venue when it closes and attend after-parties at private residences or other sex-on-premises venues, including gay saunas [28–30]. These lengthy after-parties are often facilitated by stimulant drugs and can often merge into ‘chemsex parties’ which may continue for days [30]. The extreme dehydration and exhaustion following this can exert severe mental and physical health effects [31]. Therefore, considering substance use more broadly in night-time venues, alongside the phenomenon of chemsex specifically is important.

While harm reduction strategies are well-documented and widely advocated for PWUD there remains a significant gap in addressing the specific physical contexts in which many LGBTQIA + people, particularly GBMSM, use drugs. Many existing studies focus on chemsex or harm reduction in general, without adequate attention to the unique environments where much of this substance use occurs—namely, nightclubs, bars, and sex-on-premises venues [14, 32]. These are critical sites for understanding substance use patterns and implementing harm reduction interventions [33, 34], as they are often the initial or primary settings for drug use among GBMSM [22, 25].

This study aims to explore the barriers to the uptake of harm reduction strategies among GBMSM who use drugs in LGBTQIA + nightclubs and sex-on-premises venues in London. By adopting a mixed-methods, qualitative approach, this research identifies key opportunities and challenges in promoting effective harm reduction interventions within the night-time economy, where much of this substance use occurs.

## Methods

### Inclusion criteria

Participants were over 18 years old and either identified as GBMSM with lived experience of illicit substance use in LGBTQIA + night-time venues, or as an individual with lived experience providing support for GBMSM who use illicit substances in LGBTQIA + venues. The latter group is defined as any professional role where an individual could encounter and support LGBTQIA + people who use drugs, including healthcare and commercial roles (such as security or bar staff).

### Data collection

This is a mixed-methods study combining quantitative and qualitative data from a survey and semi-structured interviews (SSIs). A survey was designed specifically for the study using insights from a literature review. Responses to survey items relating specifically to the role of night-time venues are presented for the purposes of this study. The full survey is available in *Supplementary A*. The survey was hosted on the website Qualtrics. Survey items were a series of statements with Likert-scale options to express the extent of agreement or disagreement with a given statement. An option for a neutral response was included. One open-ended question was included at the end of the survey – responses to this question have been integrated into the subsequent thematic analysis. Simple demographic data was also collected. *.*

Participants were recruited using opportunistic sampling. The survey was advertised online via social media (Instagram, LinkedIn), on posters in toilets in LGBTQIA + venues across London, and shared online by a charity involved in providing harm reduction advice for chemsex. Staff members working for the charity were also contacted by the charity internally to provide information about the study to recruit possible participants. The poster described in lay-terms that researchers were interested in surveying GBMSM with lived experience of substance use, with a QR code to the participation information sheet for additional information. An email address for the Principal Investigator was also provided. The survey was open for responses for 6 weeks in June and July 2024.

Participants could also follow a hyperlink at the end of the survey to sign up to take part in SSIs. Recruitment for these interviews was also conducted via a harm reduction charity operating in London. SSIs took place in June and July 2024. SSIs were conducted by SN. The interviewer has a background in qualitative research and works as a medical doctor specialising in psychiatry. The researcher also identifies as GBMSM and has worked with a charity providing support for individuals affected by substance use and chemsex. This interviewer was deliberately chosen to maximise the cultural competency of the interviewer, with sufficient professional experience to be able to handle sensitive conversations surrounding substance use appropriately.

Interviews were recorded onto a university laptop using conference microphone equipment and transcribed using the Microsoft Word transcription feature. Data was stored on encrypted laptops in password protected files. Only transcribed data was used for analysis. This was redacted to remove potentially re-identifying information. Each participant was given a code and referred to only by this code in the transcribed data. The interviewer was responsible for checking transcription and removing personal details. Participants were given an opportunity to review the transcript for accuracy.

### Data analysis

Data analysis was conducted by SN. Simple descriptive statistics were performed on demographic data and quantitative survey responses. Survey responses were stratified into responses from GBMSM with lived experience of substance use (GBMSM group) and those with lived experience of supporting GBMSM who use drugs (Support GBMSM group). A thematic analysis was conducted on free-text responses to the survey and SSI transcripts, following Braun & Clarke’s approach [35]. The analysis was performed inductively. SN initially screened the data to code meaningful elements, identifying overarching themes at the semantic level. SN then reviewed and refined these themes. NVivo software was used for the analysis. Triangulation across methods was achieved through various means. Trends in the quantitative data were tested with qualitative insights from SSIs and enhanced by participant lived experience to provide depth. Free-text responses from the survey were integrated into analysis of SSI transcripts. Areas of convergence or divergence between the two study groups were explored in the SSIs.

### Ethical approval

This study was reviewed and approved by the Imperial College Ethics Committee in May 2024 and granted a favourable opinion (ICREC reference number: 7045806).

## Results

The survey received a total of 53 response (GBMSM *n* = 45; support GBMSM *n* = 8). The full demographic characteristics of survey respondents is included in Table 1.

**Table 1:**
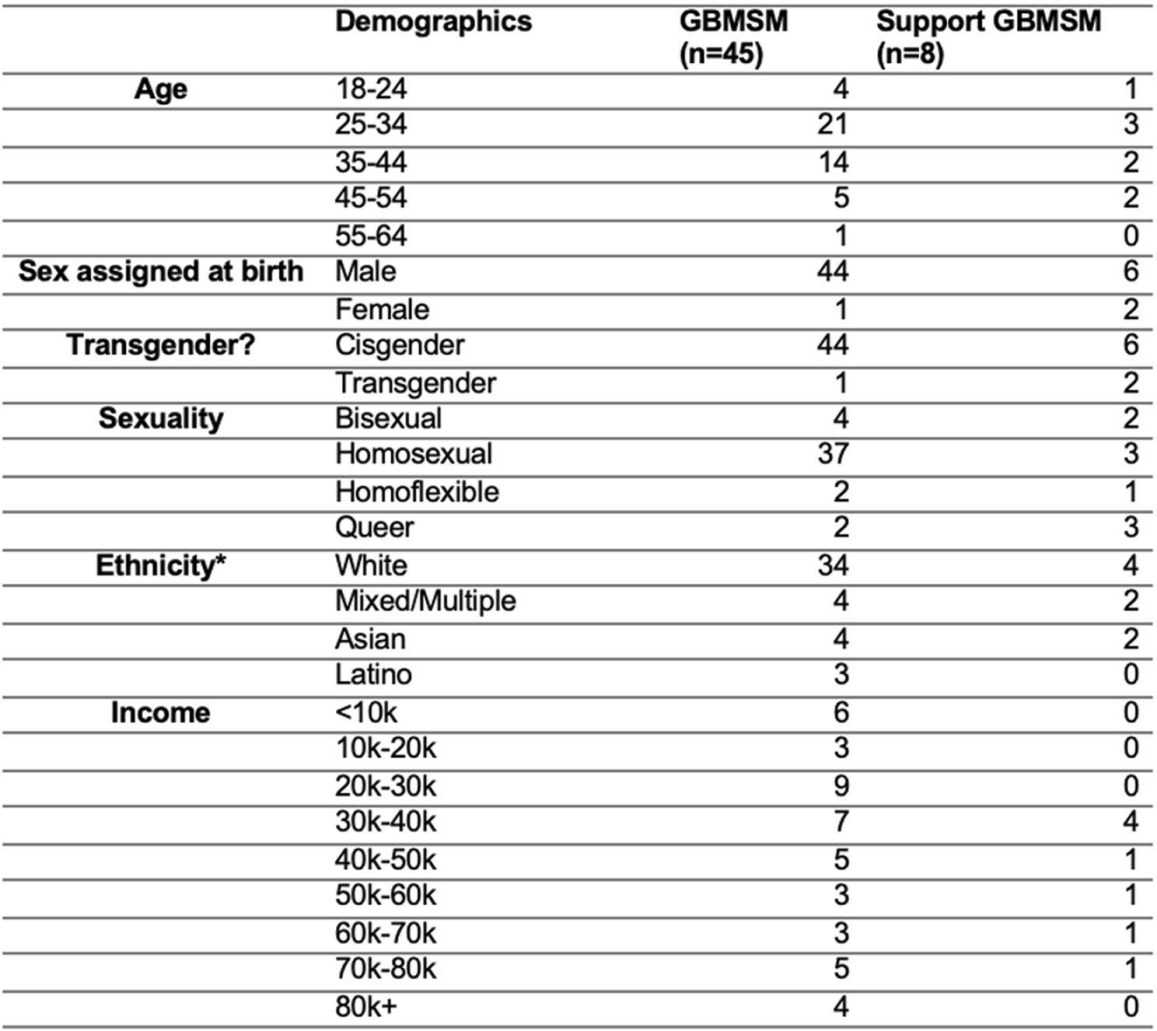
Demographics of survey participants (n=53) *Only ethnicities described by study participants have been included

SSIs were completed with an additional 8 individuals, all of whom identified as GBMSM and had used illicit substances in the past. These individuals were recruited separately to the survey respondents and therefore may not be the same individuals represented in the survey. All participants had experience receiving advice about harm reduction in relation to substance use. 4 participants had direct experience delivering harm reduction, 2 of whom delivered it in a night-club setting. Table 2 shows the full lived experience of SSI participants. Demographic data is not provided to minimise risk of possible identification.

**Table 2:**
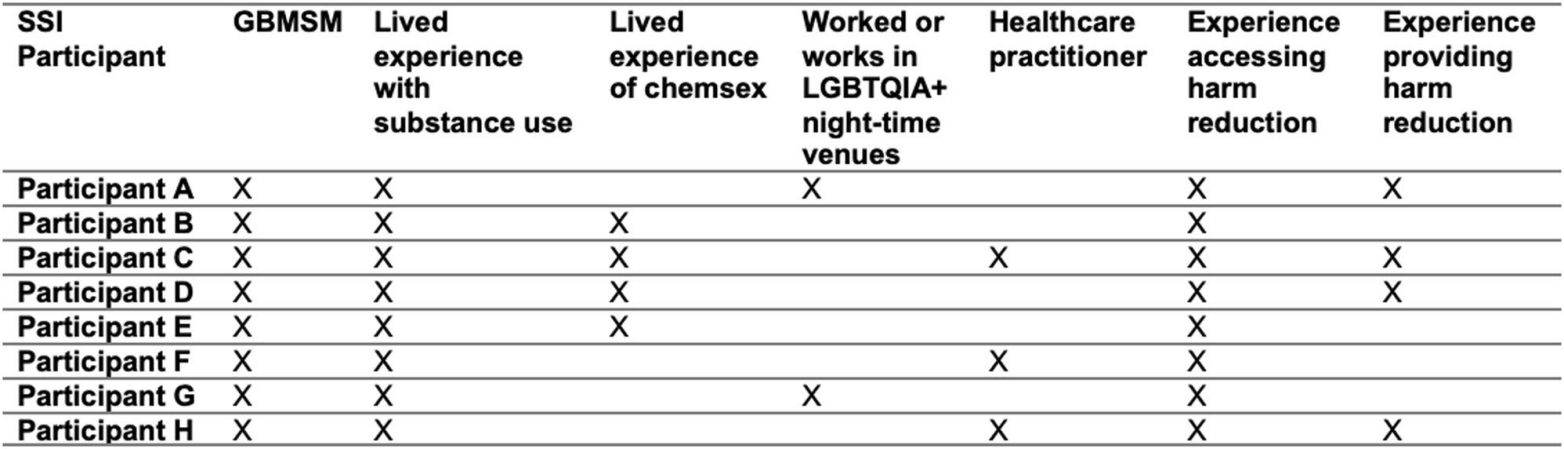
Summary of lived experience of SSI participants (n=8)

### Theme one: de facto decriminalisation versus punitive action – inconsistency across venues

**Fig. 1:**
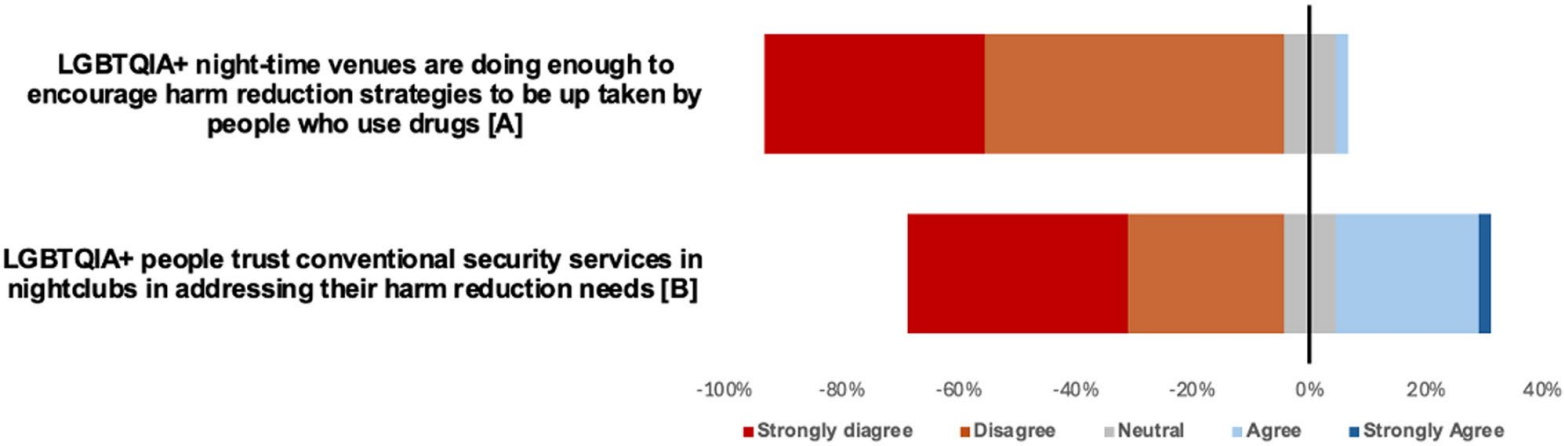
Visual representation of Likert-scale responses from GBMSM sample (*n* = 45). Data represents the number of participants who strongly disagreed to strongly agreed with the statements presented. Negative values represent the proportion of respondents who disagree or strongly disagree with the statement; positive values represent the proportion of respondents who agreed or strongly agreed with the statement. Neutral responses are equally distributed either side of the vertical line bisecting the graph at 0%. Statement A: SD 17; D 23 N 4; A 1 ; SA 0 *n* = 45). Statement B: SD 17; D 12; N 4; A 11; SA 1 (*n* = 45)

**Fig. 2:**
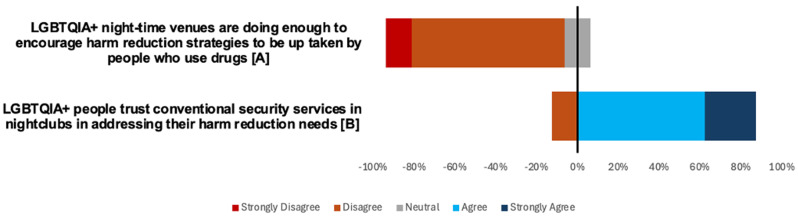
Visual representation of Likert-scale responses from ‘Staff working with GBMSM’ sample for ‘role of nighttime venues’ them (*n* = 8). Data represents the number of participants who strongly disagreed to strongly agreed with the statements presented. Negative values represent the proportion of respondents who disagree or strongly disagree with the statement; positive values represent the proportion of respondents who agreed or strongly agreed with the statement. Neutral responses are equally distributed either side of the vertical line bisecting the graph at 0%. Statement A: SD 1; D 6; N 1; A 0; SA 0. Statement B: SD 0; D 1; N 0; A 5; SA 2 (*n* = 8)

The survey results highlighted significant dissatisfaction among GBMSM with the harm reduction strategies endorsed by night-time venues. Most respondents (88.8%) disagreed or strongly disagreed that LGBTQIA + night-time venues are doing enough to promote harm reduction strategies (Fig. 1). Similarly, none of the staff surveyed agreed with this statement (Fig. 2). These findings suggest a pervasive perception of inaction or ineffective measures within these spaces. Qualitative data from the SSIs suggests inconsistency between venues could be partially driving this dissatisfaction.

Participants noted variations in harm reduction practices depending on geographic location, venue culture, and changes over time. For instance, some venues are seen to adopt a permissive stance, while others strictly enforce policies, possibly out of concern for their reputation:

*“There’s certain venues which turn a blind eye. Other venues that I’ve worked in might want to do something a bit stronger*,* but they don’t want to be seen as not being cool.” – [SSI-PARTICIPANT-A]*.

Participants also described the unintended risks created by such approaches, particularly when venues fail to address drug use openly. Surveillance measures, such as CCTV and security presence, can inadvertently push drug use into riskier, less supervised settings out of fear of negative repercussions:

*“Because of the inherent conflict between wanting to use drugs and then everything that’s out of the nightlife to stop you from using drugs. So security*,* surveillance*,* like any CCTV or surveillance*,* those sorts of things*,* sort of push people into more opportunistic or more confined spaces to use drugs that might be more risky.” – [SSI-PARTICIPANT-B]*.

This inconsistency was also reflected in temporal changes, with participants recalling a past era when venues adopted more explicit harm reduction measures, such as employing on-site medics:

“*We had clubs back in the early 00s that used to start at 4am and finish at 3pm… and all of the staff in that club knew exactly what everyone was doing. They didn’t have a problem with two people going into the cubicle. And actually*,* they had an on-site medic who would look after people if they’re in a sticky situation. I think then things changed – licensing laws got a bit stricter and there’s less of a freedom now.” – [SSI-PARTICIPANT-G]*.

### Theme two: economic and legal constraints for night-time venues

Economic and legal challenges were identified as key factors shaping harm reduction practices in night-time venues. Participants described how the need to comply with licensing laws and avoid association with drug use creates a disincentive for venues to implement harm reduction measures. This is compounded by the economic model of nightclubs, which prioritises alcohol sales over accommodating other patterns of substance use:

*“…Economically certain drugs don’t work with selling alcohol. So if a nightclub venue was to perhaps condone GHB you’re then reducing your own income from sale of alcohol.” – [SSI-PARTICIPANT-A]*.

Furthermore, strict zero-tolerance policies, often imposed to protect venues from legal repercussions, were perceived as inadvertently increasing harm by pushing drug use into less visible and riskier spaces:

*“…if you go to a gay sauna*,* probably they are not going to show that they are aware that people are doing drugs in their venues because obviously they could be shut down. In some saunas*,* they check and if they see you have GHB they give you a lifetime ban immediately.” – [SSI-PARTICIPANT-D]*.

The precarious financial position of LGBTQIA + venues further complicates harm reduction efforts, with some participants highlighting how licensing breaches could lead to closure:

*“…If you look at [REDACTED – venue name]*,* for example*,* when four or three people died*,* they had to like radically change their process of letting people in. Then if you think of the queer venues already shutting - I think like 80% shut between 2012 to now - so if they wanna stay open they need to meet these licensing restrictions*,* no matter how constrictive they are. Otherwise*,* they’ll just shut*,* so it’s quite hard.” – [SSI-PARTICIPANT-F]*.

### Theme three: nightclubs as a starting point for extended drug use and harm

Participants identified nightclubs as critical environments where patterns of extended drug use often begin, with many describing these venues as the “launching pad” for prolonged substance use that continues into informal “afters” events. The high-energy atmosphere, loud music, and social setting were seen as triggers for impulsive decision-making, with some participants pointing out how the club environment fosters a sense of invincibility:

*“You’re in the kind of party atmosphere of people laughing*,* talking*,* if you know*,* if people have already come in intoxicated or whatever*,* you don’t really have control.… it can be very impulsive when you’re there and there’s the excitement and there’s the feeling*,* you know*,* I’m invincible.” – [SSI-PARTICIPANT-H]*.

The data suggest that this disinhibition and heightened impulsivity can lead patrons to lose awareness of their limits or the substances they consume. This was particularly concerning for those who combine substances or use drugs in ways requiring specific expertise, such as injecting (“slamming”):

*“As you continue to take drugs*,* sometimes you can get more disinhibited and that’s automatically going to make you less aware of how much you’re taking*,* what exactly you’re taking*,* or where you’re going and who with.” – [SSI-PARTICIPANT-B]*.

*“It’s not just about you. It’s also about the people who you are with. And sometimes you allow people to inject*,* to slam you*,* and they don’t really know very well the techniques… But they think that they tell you*,* ‘I have done this many times*,*’ and then you tend to trust.” – [SSI-PARTICIPANT-D]*.

This relational aspect of drug use is significant. Participants described situations in which harm occurs due to misplaced trust, highlighting the importance of peer education and harm reduction messaging in nightlife settings. Furthermore, the social dynamics of nightclubs—such as peer pressure, group decision-making, and the desire to maintain the party atmosphere—can escalate risk-taking behaviours.

Participants recognised the potential for nightclubs to serve as intervention points to reduce harm, given their position at the start of a substance use trajectory. Suggested measures included the presence of trained staff or designated areas for rest and recovery:

*“It would be good to have someone who can guide them to a safe place in the club or help them get home.” – [SSI-PARTICIPANT-E]*.

Participants also indicated that while some patrons attend clubs already under the influence, a lack of explicit harm reduction measures within these spaces compounds the risks, particularly for those who may be unaware of safer substance use practices. This highlights the need for a more proactive approach by venues to integrate harm reduction strategies directly into the nightclub setting.

### Theme four: challenges in sex-on-premises venues

SOPVs were highlighted as particularly high-risk environments for harm, not only due to the prevalence of drug use but also because of the unique practices and policies in these spaces. Participants described zero-tolerance policies as a major barrier to effective harm reduction. These policies, implemented to protect the venues from legal repercussions, often drive individuals to pre-load with drugs before entering the venue, leading to dangerously high levels of intoxication:

*“…They [PWUD] just take it all just at once when they take off their clothes when they arrive. You know what I mean? And then sometimes they come into harm that way because they’re just massively high.” – [SSI-PARTICIPANT-B]*.

Participants also identified spiking as a growing concern in these venues. The covert administration of substances, such as GHB, can occur through unconventional means, like laced lubricants, posing significant risks to patrons unaware of the dangers:

*“Some people are spiking others [in SOPVs]. There are people who want to rape someone… they go to the darkroom or the sauna or over to their house and they put GHB in the lube*,* and obviously the membranes there are very thin… So overdoses are happening because people are not expecting this.” – [SSI-PARTICIPANT-D]*.

The design of SOPVs further complicates harm reduction. Darkrooms, private areas, and the intimate nature of these spaces can make it difficult for staff to monitor drug use or intervene effectively during crises. Participants shared examples of incidents involving overdoses and unsafe behaviours, underscoring the urgent need for tailored harm reduction strategies:

*“[SOPVs are aware that]… There’s so many examples of people that we’ve lost through ways it can go wrong… in psychosis from crystal meth and jumping off a balcony*,* or drowning in a jacuzzi because they take too much GHB*,* the list could go on…” – [SSI-PARTICIPANT-A]*.

Some participants pointed to harm reduction models in European countries, such as the Netherlands, where drug testing services and educational initiatives are integrated into nightlife settings. These approaches were viewed as potential solutions to the challenges faced by SOPVs in the UK:

*“…If we look at Europeans*,* European set-ups especially*,* maybe the Netherlands and stuff*,* they will offer drug testing at raves and harm reduction.” – [SSI-PARTICIPANT-H]*.

Overall, the findings highlight a tension between the zero-tolerance policies implemented to maintain safety and the unintended harms these policies create. Participants called for a more pragmatic approach, balancing legal and economic considerations with evidence-based harm reduction strategies tailored to the specific needs of these venues.

## Discussion

This mixed-methods study highlights the potential role of night-time venues, including nightclubs and SOPVs, in promoting harm reduction strategies for GBMSM who use drugs. Our findings underscore the potential of these spaces to address harms beyond sexual health, with particular focus on mental health and substance-related risks.

To our knowledge, this is the first study of its kind to combine the perspectives of GBMSM who use drugs, those involved in supporting GBMSM who use drugs, and the perspective of individuals working in the night-time economy in relation to harm reduction. Previous research has analysed night-clubs and SOPVs primarily through a sexual health lens, focussing on risks such as sexually transmitted infections (STIs) [33, 34]. Indeed, there is long-standing evidence of higher diagnosed rates of syphilis and other STIs in people attending commercial SOPVs [36]. Moreover, there is an over-representation of people living with HIV (PLWH) and risky sexual behaviours, including not using condoms, in people attending SOPVs [28, 36, 37]. In contrast, our study indicates a relative lack of focus on the mental health components of harm reduction for substance use - including chemsex – in such settings, which is consistent with existing literature [38, 39]. This finding suggests that healthcare providers working beyond sexual health settings, particularly general practitioners and psychiatrists, should be more prepared to discuss substance use and specifically chemsex with their GBMSM patients [38].

Participants consistently reported the perception that nightclubs and SOPVs could do significantly more to promote harm reduction strategies. This reflects a critical gap in current practice, particularly when compared to historical public health interventions. For example, during the HIV/AIDS crisis, night-time venues were instrumental in the widespread rollout of condoms which became a cornerstone of efforts to reduce sexual health risks within LGBTQIA + communities [40, 41]. A similar approach could now be applied to address the harms associated with chemsex and substance use. Peer-led education and support has also been demonstrated to be a possible vehicle to promote harm reduction [42]. Night-time venues could provide the appropriate social opportunity to commence these discussions and enhance peer-to-peer learning [43]. If night-time venues were to integrate harm reduction resources, such as drug safety information, overdose prevention tools, and staff trained in recognising and responding to substance-related emergencies, they could play a pivotal role in mitigating risks not only within their walls but also beyond them.

A key theme emerging from the data is the link between nightclubs and ‘afters’ culture. These informal gatherings, which often occur in private settings following the closure of official venues, were identified as high-risk environments where substance use is frequently prolonged and poorly monitored [30]. Participants described how behaviours that begin in structured spaces like nightclubs often escalate during these poorly monitored ’afters‘ events, where there is limited access to harm reduction resources or support [30]. The stimulating and high-energy atmosphere of nightclubs was also noted to foster impulsive decision-making, potentially contributing to escalated substance use later in the night. This highlights the potential for interventions initiated in nightclubs to filter into the broader behavioural landscape of their patrons [43]. For instance, the distribution of drug-testing kits, provision of information about safer substance use practices, or even basic overdose prevention tools in nightclub settings could empower individuals to carry these resources into informal settings. Such measures might reduce the harms associated with ‘afters’ culture, creating a ripple effect of harm reduction practices that extend beyond the immediate nightclub environment. However, this would need to be explored in follow-up studies.

However, the inconsistent approach to substance use and harm reduction in night-time venues reflects a broader challenge within the regulatory and economic environment. The current 10-Year Drugs Strategy in the United Kingdom maintains a predominantly punitive stance towards substance use, which has drawn criticism from some stakeholders [44, 45]. Many venues operate under the constant threat of punitive enforcement policies, discouraging them from openly engaging with harm reduction strategies. Our study suggests this zero-tolerance stance not only stifles the potential for venues to address substance use proactively but also creates unintended risks for patrons. For example, in SOPVs, participants described behaviours such as preloading substances or using them covertly inside venues to avoid detection. Such practices can increase the likelihood of overdose, adverse drug interactions, or other health emergencies.

**Fig. 3:**
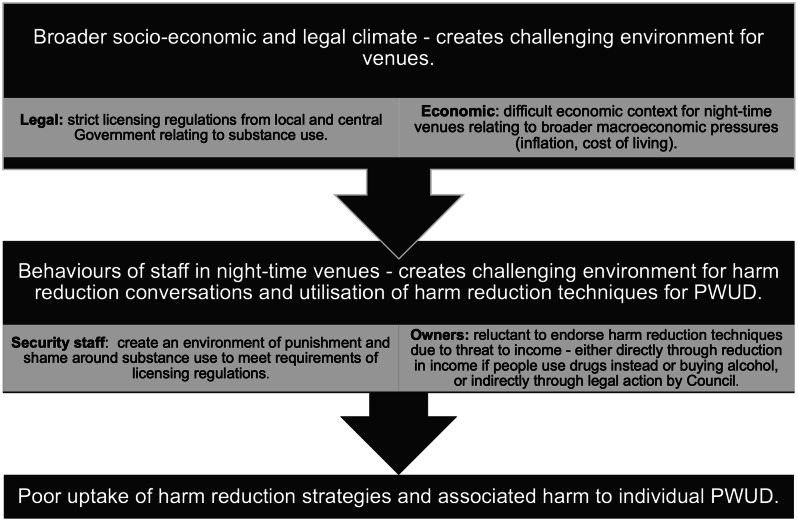
A simplified schematic showing the mediator role that night-time venues can play in perpetuating drug-related harm as a middle-ground between the Government and individual PWUD

Figure 3 illustrates how nightclubs and other night-time venues mediate between governmental policies and individual behaviours, occupying a pivotal middle ground in the harm reduction landscape. On one side, these venues are constrained by restrictive licensing laws and punitive drug policies; on the other, they engage directly with communities where substance use is prevalent. This unique positioning enables venues to act as structured environments where harm reduction practices could be normalised, provided systemic barriers such as licensing constraints are addressed. Legal reforms, such as pilot schemes for safer consumption spaces or amendments to licensing laws that permit venues to distribute harm reduction materials, could enable night-time venues to adopt these strategies without jeopardising their operational viability.

### Limitations

This study has important limitations impacting the external validity. The sample size recruited for the survey is small and insufficiently powered to analyse intersecting demographics that may act as confounders for uptake of harm reduction techniques. Whilst saturation of themes was reached, it would be beneficial to recruit a higher number of individuals with experience working in night-time venues. There is also a risk of selection bias by recruiting participants through LGBTQIA + night-time venues and a harm reduction charity, however this likely contributed to the rich discussion found in the SSIs. The use of one person to identify and code themes is another limitation, but the use of mixed methods to test emerging trends mitigates this somewhat.

## Conclusion

This study considers the role of night-time venues – including night-clubs and SOPVs– as a barrier to utilisation of harm reduction techniques amongst GBMSM. Currently, there is a perception among people with lived experience of substance use that night-time venues could do more to encourage use of harm reduction techniques. These findings underline the untapped potential of night-time venues to address the evolving public mental health needs of GBMSM who use drugs. While historically focused on reducing sexual health risks, these spaces could now play a transformative role in mitigating mental health and substance-related harms. By encouraging adoption harm reduction strategies, night-time venues can shift from being merely recreational spaces to becoming proactive allies in reducing the broader risks associated with substance use and chemsex within their communities. This shift not only addresses an urgent public mental health need but also reinforces the social and cultural significance of these spaces in promoting the well-being of GBMSM.

## Electronic supplementary material

Below is the link to the electronic supplementary material.

Supplementary Material 1

## Data Availability

Data is provided within the manuscript or supplementary information files.
