# Supplementary material for: Barriers to uptake of harm reduction techniques for GBMSM who use drugs in night-clubs and sex-on-premises venues in London and the Southeast: a mixed-methods, qualitative study
Source: Harm Reduct J. 2025 Feb 1;22:13. doi: 10.1186/s12954-025-01159-2 (PMC11786357; doi:10.1186/s12954-025-01159-2)
Supplement: Supplementary file 1 — Supplementary Material 1 [file 12954_2025_1159_MOESM1_ESM.docx]

**SUPPLEMENTARY A**

**SURVEY DESIGN**

**Demographics:**

**Age**

- 18-24

- 25-34

- 35-44

- 45-54

- 55-64

- 65+

**What was your sex assigned at birth?**

- Female
- Male
- Don’t know
- Prefer not to say

**Is your gender the same as the sex you were assigned at birth?**

- Yes
- No – please specify your gender identity
- Prefer not to say

**Please describe your sexual orientation.***

- Lesbian
- Gay
- Bisexual
- Pansexual
- Queer
- Asexual
- Demisexual
- Homoflexible
- Heteroflexible
- Other/I would like to describe my sexual orientation in my own words: (please specify)

[*Note: the use of such a wide range of options and the ability to self-describe is an effort to be LGBTQIA-identity affirming. The researcher did not want to dissuade participants from taking part by appearing culturally insensitive].

**Please tick the box which describes your ethnicity.**

- White/Caucasian

- Black/African American

- Hispanic/Latinx

- Asian/Pacific Islander

- Native American/Indigenous

- Middle Eastern/North African

- Mixed/Multiracial

- Other/I would like to describe my ethnicity in my own words: (please specify)

**Income:**

- Less than £10,000

- £10,001-£20,000

- £20,001-£30,000

- £30,001-£40,000

- £40,001-£50,000

- Over £50,000

**Professional group:**

- Full-time employed
- Part-time employed
- Self-employed
- Unemployed
- Student
- Retired
- Other (please specify)

**What is the highest level of education you have undertaken?**

- No formal qualifications

- GCSEs/O Levels or equivalent

- A Levels or equivalent

- Vocational qualification (e.g., NVQ, BTEC)

- Bachelor's degree

- Master's degree

- Doctoral degree

- Professional qualification (e.g., ACCA, CIMA)

- Other (please specify)

**Which role most accurately describes your reason for completing this survey?**

- Professional working in harm reduction
- Attendee at LGBTQIA+ night-time venues

[SURVEY SPLITS INTO TWO FOR EACH STAKEHOLDER RESPECTIVELY]

**PRIMARY STAKEHOLDER: PROFESSIONAL WORKING IN HARM REDUCTION**

**Please select all harm reduction methods you have seen utilised in LGBTQIA+ venues:**

- [ ] Needle exchange programs

- [ ] Condom distribution programs

- [ ] Access to naloxone or overdose reversal medication

- [ ] Educational programs on safer drug use

- [ ] HIV/STI testing services

- [ ] Substance use counselling or therapy

- [ ] Access to mental health support services

- [ ]On-site welfare teams and/or first aid

- [ ] Other (please specify): ____________

**For the following, please select from ‘strongly disagree’ to ‘strongly agree’:**

Experiences with healthcare settings:

1. LGBTQIA+ people face challenges or barriers when trying to access harm reduction services in healthcare settings. (*SD, D, N, A, SA)*
2. LGBTQIA+ people trust conventional healthcare services in addressing my harm reduction needs. (*SD, D, N, A, SA)*
3. LGBTQIA+ people trust conventional security services in addressing my harm reduction needs. (*SD, D, N, A, SA)*
4. LGBTQIA+ people are satisfied with the cultural competency of healthcare providers in harm reduction services. (*SD, D, N, A, SA)*
5. LGBTQIA+ people would prefer to access specialised LGBTQIA+ harm reduction services rather than mainstream services. (*SD, D, N, A, SA)*

Education on harm reduction

1. Enough is being done to educate LGBTQIA+ people about harm reduction strategies. (*SD, D, N, A, SA)*
2. LGBTQIA+ night-time venues are doing enough to encourage harm reduction strategies to be up taken by people who use drugs. (*SD, D, N, A, SA)*
3. Mainstream healthcare settings sufficiently understand the unique needs of LGBTQIA+ people in relation to harm reduction. (*SD, D, N, A, SA)*
4. The current UK policy towards substance use is sufficient to minimise harm to people who use drugs. (*SD, D, N, A, SA)*

Chemsex Awareness:

1. I am aware of some of the concept of chemsex. (*SD, D, N, A, SA)*
2. I am aware of harm reduction techniques one can utilise during chemsex. (*SD, D, N, A, SA)*
3. I have supported LGBTQIA+ people who have encountered barriers when trying to access harm reduction advice on chemsex specifically. (*SD, D, N, A, SA)*

**For the following section, please provide written answers to the following questions:**

1. What do you feel the main barriers are to improving uptake of harm reduction advice for LGBTQIA+ people who use drugs? [TEXT BOX]
2. What would encourage LGBTQIA+ people to engage with the harm reduction services you offer? [TEXT BOX]
3. How can we create a safer and more inclusive approach towards harm reduction? [TEXT BOX]
4. What changes in policies would you personally recommend to better support harm reduction initiatives? [TEXT BOX]

**SECONDARY STAKEHOLDER: ATTENDEE AT LGBTQIA+ VENUES**

**Please select all harm reduction methods you have personally used:**

- [ ] Needle exchange programs

- [ ] Condom distribution programs

- [ ] Access to naloxone or overdose reversal medication

- [ ] Educational programs on safer drug use

- [ ] HIV/STI testing services

- [ ] Substance use counselling or therapy

- [ ] Access to mental health support services

- [ ] On-site welfare teams and/or first aid

- [ ] Other (please specify): ____________

**For the following, please select from ‘strongly disagree’ to ‘strongly agree’:**

Experiences with healthcare settings:

1. I have faced challenges or barriers when trying to access harm reduction services in healthcare settings. (*SD, D, N, A, SA)*
2. I trust conventional healthcare services in addressing my harm reduction needs. (*SD, D, N, A, SA)*
3. I trust conventional security services in addressing my harm reduction needs. (*SD, D, N, A, SA)*
4. I am satisfied with the cultural competency of healthcare providers in harm reduction services. (*SD, D, N, A, SA)*
5. I would prefer to access specialised LGBTQIA+ harm reduction services rather than mainstream services. (*SD, D, N, A, SA)*

Education on harm reduction

1. Enough is being done to educate LGBTQIA+ people about harm reduction strategies. (*SD, D, N, A, SA)*
2. LGBTQIA+ night-time venues are doing enough to encourage harm reduction strategies to be up taken by people who use drugs. (*SD, D, N, A, SA)*
3. Mainstream healthcare settings sufficiently understand the unique needs of LGBTQIA+ people in relation to harm reduction. (*SD, D, N, A, SA)*
4. The current UK policy towards substance use is sufficient to minimise harm to people who use drugs. (*SD, D, N, A, SA)*

Chemsex Awareness:

1. I am aware of some of the concept of chemsex. (*SD, D, N, A, SA)*
2. I am aware of harm reduction techniques one can utilise during chemsex. (*SD, D, N, A, SA)*
3. I have encountered barriers when trying to access harm reduction advice on chemsex specifically. (*SD, D, N, A, SA)*

**For the following section, please provide written answers to the following questions:**

1. What do you feel the main barriers are to improving uptake of harm reduction advice for LGBTQIA+ people who use drugs? [TEXT BOX]
